# Can posterior stand-alone expandable cages safely restore lumbar lordosis? A minimum 5-year follow-up study

**DOI:** 10.1186/s13018-020-01866-5

**Published:** 2020-09-29

**Authors:** Seung-Kook Kim, Ogeil Mubarak Elbashier, Su-chan Lee, Woo-Jin Choi

**Affiliations:** 1 Himchan and UHS Spine and Joint Centre, University Hospital Sharjah, Sharjah, United Arab Emirates; 2 Department of Pharmaceutical Medicine and Regulatory Sciences, College of Medicine and Pharmacy, Yonsei University, Seoul, Republic of South Korea; 3 Joint and Arthritis Research, Orthopaedic Surgery, Himchan Hospital, Seoul, Republic of South Korea; 4 Department of Spine Center, Neurosurgery, Hurisarang Hospital, 618 Gyeryong-ro, Seo-gu, Daejeon, 35299 Republic of South Korea

**Keywords:** Lumbar lordosis, Proximal junctional kyphosis, Sacral slope, Sagittal imbalance, Stand-alone expandable cage fusion

## Abstract

**Background:**

Lumbar lordosis (LL) can be restored, and screw-related complications may be avoided with the stand-alone expandable cage method. However, the long-term spinopelvic changes and safety remain unknown. We aimed to elucidate the long-term radiologic outcomes and safety of this technique.

**Methods:**

Data from patients who underwent multi-level stand-alone expandable cage fusion and 80 patients who underwent screw-assisted fusion between February 2007 and December 2012, with at least 5 years of follow-up, were retrospectively analyzed. Segmental angle and translation, short and whole LL, pelvic incidence, pelvic tilt, sacral slope (SS), sagittal vertical axis, thoracic kyphosis, and presence of subsidence, pseudoarthrosis, retropulsion, cage breakage, proximal junctional kyphosis (PJK), and screw malposition were assessed. The relationship between local, lumbar, and spinopelvic effects was investigated. The implant failure rate was considered a measure of procedure effectiveness and safety.

**Results:**

In total, 69 cases were included in the stand-alone expandable cage group and 150 cases in the control group. The stand-alone group showed shorter operative time (58.48 ± 11.10 vs 81.43 ± 13.75, *P* = .00028), lower rate of PJK (10.1% vs 22.5%, *P* = .03), and restoration of local angle (4.66 ± 3.76 vs 2.03 ± 1.16, *P* = .000079) than the control group. However, sagittal balance (0.01 ± 2.57 vs 0.50 ± 2.10, *P* = .07) was not restored, and weakness showed higher rate of subsidence (16.31% vs 4.85%, *P* = .0018), pseudoarthrosis (9.92% vs 2.42%, *P* = .02), cage, and retropulsion (3.55% vs 0, *P* = .01) than the control group.

**Conclusions:**

Stand-alone expandable cage fusion can restore local lordosis; however, global sagittal balance was not restored. Furthermore, implant safety has not yet been proven.

## Background

With the global aging society, degenerative lumbar spine disease is becoming a common health issue. Degenerative lumbar spine disease not only causes spinal stenosis but is also related to structural and functional problems. One such problem, sagittal imbalance, is a crucial contributing factor to a decreased quality of life [1, 2].

Various approaches have been investigated to restore lumbar lordosis (LL) and sagittal balance. Direct decompression and fusion methods, including posterior lumbar interbody fusion and transforaminal lumbar interbody fusion, can achieve both canal decompression and solid fusion; nonetheless, the invasiveness into the bone and musculature is a drawback [3]. With the indirect decompression method, anterior lumbar interbody fusion [4] and lateral lumbar interbody fusion [5] can achieve a greater lordotic curve than that via the posterior approach; however, the possibility of incomplete decompression is a shortcoming.

Stand-alone expandable cages have been designed for restoring LL and correcting sacropelvic imbalance with simultaneous canal decompression, and these cages help avoid screw-related complications. Stand-alone cage showed efficacy in improving clinical symptom and a high fusion rate in a case series [6], and an expandable cage showed efficacy in restoring the sagittal balance in a retrospective analysis [7]. To the best of our knowledge, the long-term outcomes and safety of this procedure have not been established to date. Lumbar interbody fusion with screw fixation is the most common procedure for stabilization of spinal segment [3, 5], but it was not compared with a stand-alone technique. Thus, in this study, we hypothesized that if stand-alone expandable cages can restore and maintain sagittal balance safely; it can be an effective procedure. This study aimed to elucidate the long-term radiologic efficacy of this technique and evaluate its safety with implant issues.

## Methods

### Study design and population

This retrospective study reviewed prospective cohort medical data and radiographic findings of patients consecutively treated between February 2007 and December 2012 in a single spine institute. This study was conducted in accordance with the principles outlined in the Declaration of Helsinki and was approved by the institutional review board (Himchan IRB 169684-01-201906-04); furthermore, written informed consent was obtained from all patients. This manuscript adheres to the STROBE recommendations for reporting observational studies.

**Fig. 1:**
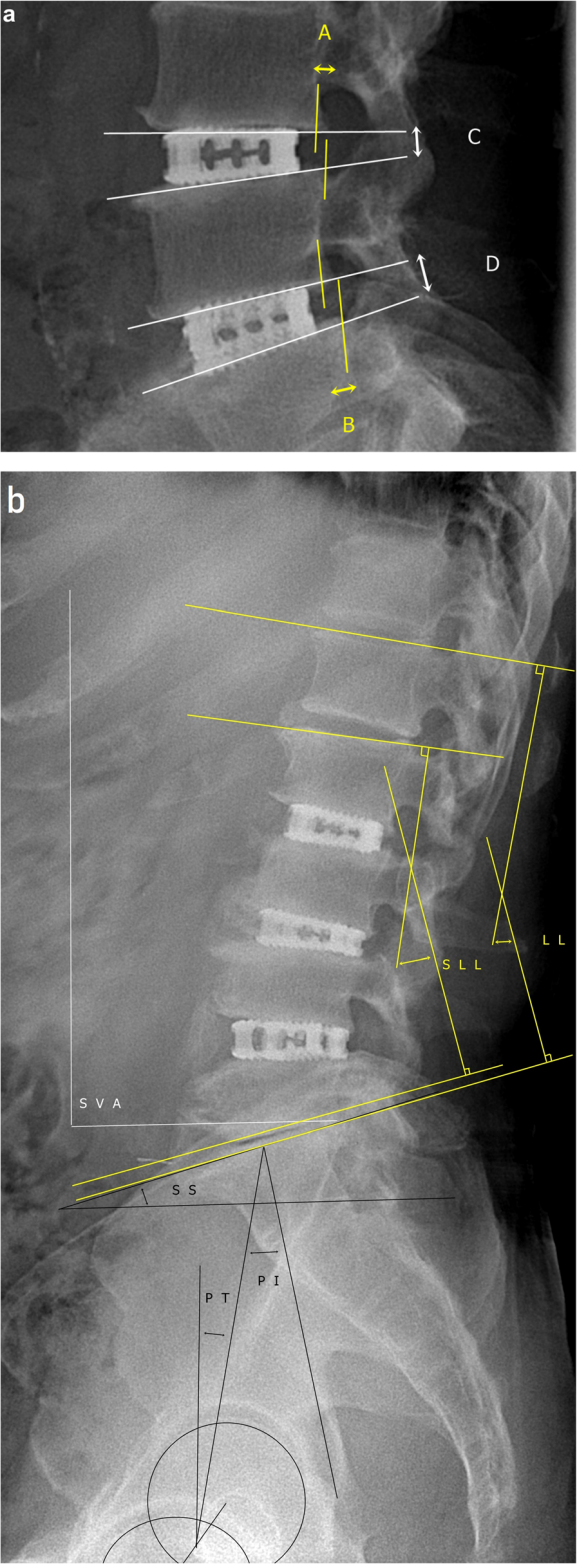
Radiologic parameters. **a** Local factors. Translation is calculated as the mean of the diameters of translation (yellow characters). Segmental angle is calculated as the mean of the Cobb angles (white characters). **b** Lumbar factors. Lumbar lordosis (yellow characters) and spinopelvic profile (white characters). PI, pelvic incidence; PT, pelvic tilt; SLL, segmental lumbar lordosis; SS, sacral slope; SVA, sagittal vertical angle; WLL, whole lumbar lordosis

The study population selection is shown in Fig. 1. The minimum sample size was calculated using G-power for Windows software (version 3.1.9.4; Brunsbüttel, Germany). Because the safety and efficacy of single-level sagittal restoration is controversial [8] and proximal junctional kyphosis (PJK) shows different sagittal and spinopelvic profiles [9], these conditions were excluded. Among 1088 cases of fusion surgery, the following inclusion criteria were applied: (1) degenerative lumbar disease symptoms present for > 2 months, (2) spinal instability confirmed on dynamic radiography but spondylolisthesis grade I or II only, and (3) involvement of at least two spinal levels. The exclusion criteria were (1) other causes of deformity (e.g., adolescent idiopathic scoliosis, PJK), (2) presence of a tumor, (3) infection, or (4) trauma.

### Operative technique and in-hospital management

In most cases, we applied a facet-preserving technique [10] to support interbody fusion. To preserve sagittal stability before a fixed interbody fusion, more than 50% of the facet was preserved. The surgery was performed after spinal or epidural anesthesia. After applying an aseptic operative field dressing with alcohol and betadine, spinal levels were checked with a c-arm before incisions were made. After a midline incision was made based on the operative level, both multifidus muscles were dissected with a monopolar coagulator. Laminectomy was performed with a high-speed air drill and Kerrison punches, and the lower half of the upper laminar, lower half of the spinous process, and upper half of the lower laminar were removed. The ligamentum flavum was removed after the bone and ligament junction were detached with curettes. After meticulous bleeding control in the disc space, total discectomy was performed with a knife, pituitary forceps, and shavers. Rotating types of 8–10° expandable interbody cages were used (Varian^TM^, Medyssey Co., Jecheon, Korea) for cage implantation. After fluoroscopic confirmation with the c-arm, muscle, and skin were sutured layer by layer.

Patients had 3 days of bed rest, and radiography was performed on postoperative day (POD) 3 and POD 15. Patients were fitted with a thoracolumbar sacral orthosis to be used as a brace for 2 months post-surgery.

In the control group, the same laminectomy and discectomy procedures were performed; subsequently, a polyetheretherketone cage was inserted bilaterally, and pedicular screws and lordotic curved rods were applied. Ambulation started on POD 1, and radiography was performed on POD 1 and POD 10. The control group was also fitted with a thoracolumbar sacral orthosis to be used as a brace for 2 months post-surgery.

After procedure, both groups were prescribed intravenous patient-controlled analgesia, acetaminophen, intermittent neuroleptics, and opioids. Blood test results were checked for inflammation on POD 3 and 7. Outpatient clinic follow-up was schedules after 2 weeks, 1 month, 6 months, and yearly.

### Radiographic assessment

The radiographic variables used in this study are shown in Table 1. Segmental parameters, such as the segmental angle and translation, were checked [14] to evaluate the local effect (Fig. 1a). For the evaluation of LL, the short LL, and whole LL were checked [15]. The following spinopelvic parameters were evaluated: pelvic incidence (PI), pelvic tilt (PT), and sacral slope (SS) (Fig. 1b) [14]. Global sagittal balance was assessed based on sagittal vertical balance, and thoracic kyphosis was assessed using a compensation mechanism [16]. Fusion rate and implant failures included subsidence [17], pseudoarthrosis, retropulsion, cage breakage, PJK, and screw malposition [18]. All variables were assessed after 1 year and at the final follow-up. All data were measured by three different observers (SKK, OME, and WJC) who had all worked as spinal physicians for more than 10 years.

**Table 1:** Descriptions of the measurements and implant failure

Category	Parameter	Definition
Local factor	Segmental angle [11]	Angle between the perpendicular line of the lower endplate of the upper vertebra and upper endplate of the lower vertebra
	Segmental translation [11]	Forward or backward slippage on a lateral radiograph
Lumbar factor	Short lumbar lordosis [5]	Cobb angle between the upper endplate of the fused vertebra and lower endplate of the fused vertebra
	Whole lumbar lordosis [5]	Cobb angle between the upper endplate of L1 and the lower endplate of L5
Spinopelvic factor	Pelvic incidence [12]	Angle between the perpendicular line to the mid-point of the upper sacral endplate and mid-point of both femoral heads
	Pelvic tilt [12]	Angle between the vertical line from the femoral head and center of the sacral endplate
	Sacral slope [12]	Angle between the vertical line and superior sacral endplate
Global sagittal balance	Thoracic kyphosis [12]	Angle between the T4 upper endplate and T12 lower endplate
	Sagittal vertical axis [12]	Distance from the vertical line of the C7 body to the inferior lateral corner of the L5 body
	Sagittal balance [12]	Sagittal vertical axis line located within 5 cm
Implant failure	Subsidence [13]	Greater or equal to 2 mm loss of height
	Pseudoarthrosis [6]	Bony non-union between two vertebrae
	Proximal junctional kyphosis [6]	Proximal junction Cobb angle of at least 10° greater than the preoperative angle
	Screw malposition [6]	Perforated pedicular screw

### Statistical analysis and proficiency matching

Statistical analyses were performed using R software for Windows version 3.6.1 (R Foundation for Statistical Computing, Vienna, Austria). A *P* value of less than .05 was considered statistically significant. Continuous variables (age, operative time, hospital stay, and all radiologically measured angles and lengths) were compared using the unpaired Student’s *t* test. Categorical variables (sex, operative type, and preoperative medical grade) were compared using the chi-square test. Proficiency matching between the segmental angle, LL, PI, and sagittal vertical balance was performed using the Pearson correlation analysis to identify whether positive correlation exists between local and global factors.

## Results

### Baseline demographics

**Fig. 2:**
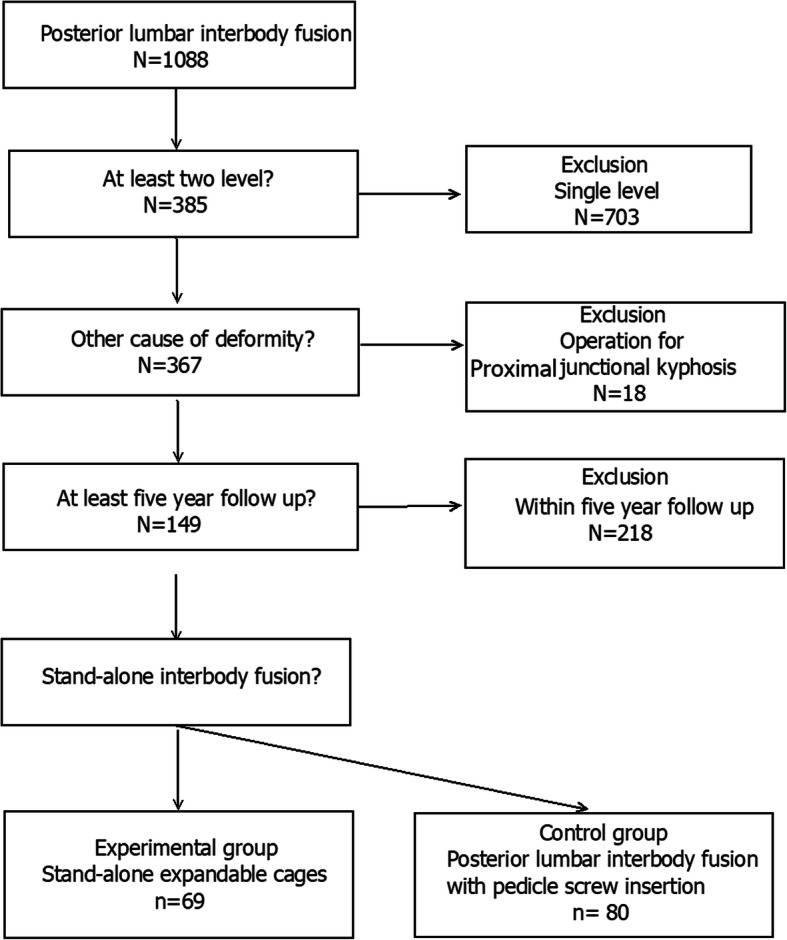
Study population selection

The study population selection is described in Fig. 2. Of the 385 remaining cases after the application of the inclusion and exclusion criteria, single-level cases (*n* = 703), fusion for PJK cases (*n* = 18), and cases with short follow-up duration (*n* = 235) were excluded. The final patient group (*n* = 149) was divided into an experimental group (*n* = 69) who received an expandable cage and a control group (*n* = 80).

Demographic data are summarized in Table 2. Age, sex, duration of symptoms, follow-up duration, and preoperative medical condition did not significantly differ between the two groups. The majority of surgeries performed involved two spinal levels, as opposed to three levels, in both groups (cage-alone, 95.66%; control, 93.75%; *P* = .45). The mean operative time was shorter in the cage-alone group than in the control group (58.48 min/level vs 81.43 min/level; *P* = .00028). Hospital stay was longer in the cage-alone group than in the control group (14.10 days vs 10.12 days; *P* = .00001).

**Table 2:** Baseline characteristics of participants

Factor	Total (***n*** = 149)	Stand-alone expandable cage fusion (***n*** = 69)	Screw-assisted fusion (***n*** = 80)	***P*** value
Age (years), mean (SD)	63.25 (8.37)	61.78 (8.05)	64.51 (8.50)	.15^a^
Sex (%)				.36^b^
Male	38 (25.5)	M: 19 (27.54)	M: 19 (23.75)	
Female	111 (74.5)	F: 50 (72.46)	F: 61 (76.25)	
Symptom duration (months), mean (SD)	9.52 (10.40)	10.86 (12.74)	8.36 (7.75)	.14^a^
Follow-up duration (months), mean (SD)	72.91 (17.53)	75.23 (14.23)	70.91 (11.62)	.10^a^
ASA-PS grade, %, mean (SD)				.82^b^
1	13 (8.72)	4 (10.14)	6 (75)	
2	144 (87.92)	67 (86.96)	77 (88.75)	
3	5 (3.36)	69 (2.9)	1 (3.75)	
No. of involved levels, %, mean (SD)				.45^b^
2	141 (94.63)	66 (95.65)	75 (93.75)	
3	8 (5.37)	3 (4.35)	5 (6.25)	
Operative time/level, min, mean (SD)	70.80 (17.01)	58.48 (11.10)	81.43 (13.75)	.00028^a^*
Hospital stay, days, mean (SD)	11.96 (2.85)	14.10 (1.97)	10.12 (2.10)	.00001^a^*

^a^Independent Student’s *t* test

^b^ *χ*^2^ test

*P < .05

*ASA-PS* American Society of Anesthesiologists physical status, *F* female, *M* male, *No.* number, *SD* standard deviation

### Radiologic outcomes

**Fig. 3:**
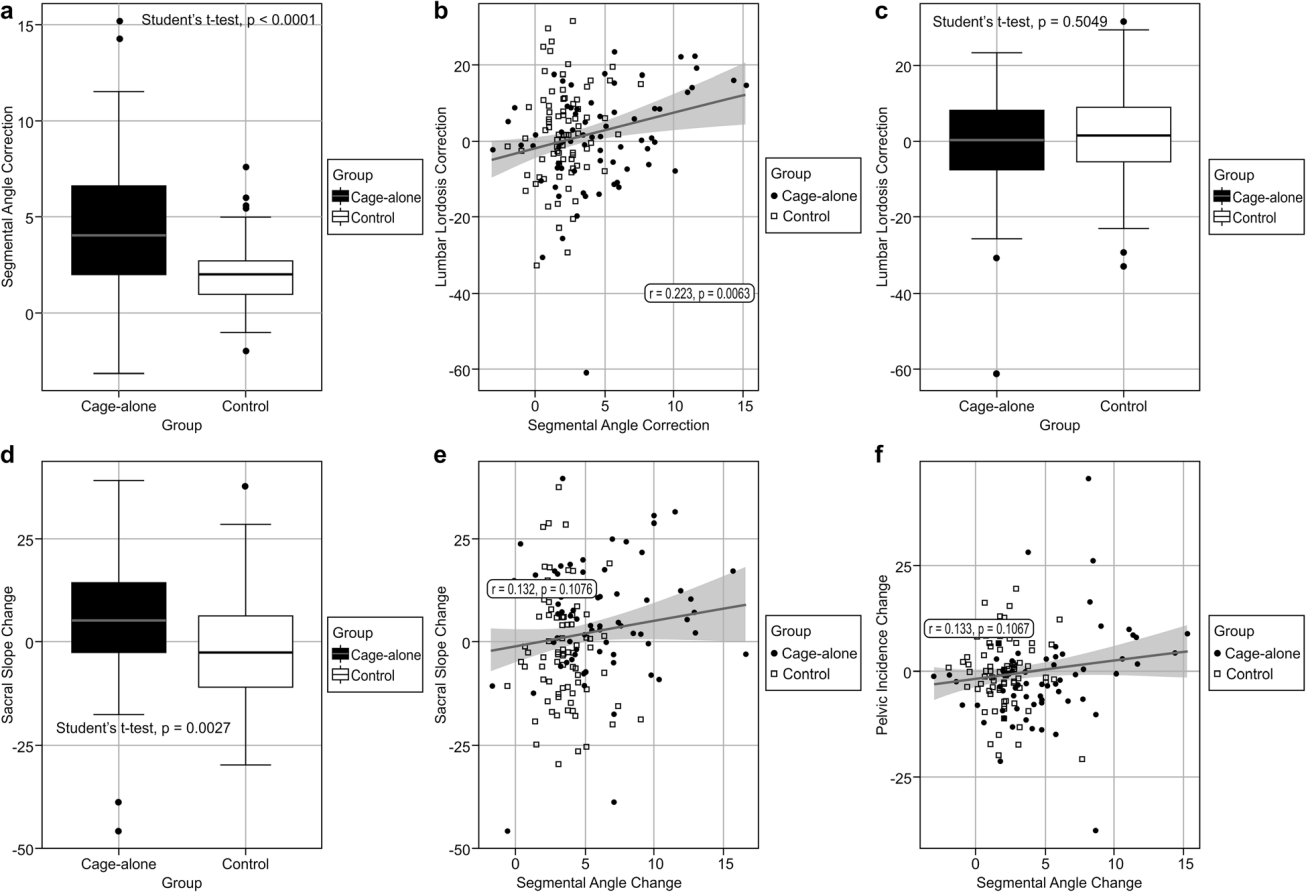
Relationship between sacropelvic profiles. **a** Comparison of the mean segmental corrections. **b** Correlation between the segmental angle change and lumbar lordosis. **c** Comparison of the mean lumbar lordosis corrections. **d** Comparison of the mean sacral slope changes. **e** Correlation between the segmental angle change and sacral slope change. **f** Correlation between the segmental angle change and pelvic incidence change

Evaluation of the preoperative, short-term, and final radiologic outcomes is summarized in Table 3. With respect to the comparison of local and lumbar factors, the segmental angle was significantly corrected (*P* = .000079; Fig. 3a). The segmental angle and short LL were greatly corrected as shown in the short-term results, and the correction angle decreased in both groups at the final follow-up. The segmental angle and LL showed a significant positive correlation in the Pearson correlation analysis (Fig. 3b) (*r* = .223, *P* = .0063). However, this segmental lordosis correction did not restore LL (*P* = .5049) (Fig. 3c). Even though SS significantly increased (*P* = .0027) (Fig. 3d), PT decreased; as a result, PI was not significantly changed. Overall, global sagittal balance did not significantly change in either group.

**Table 3:** Comparison of the preoperative, 1 year, and final follow-up radiologic parameters in each group

	Stand-alone expandable cage fusion (***n*** = 69)				Screw-assisted fusion (***n*** = 80)				***P*** value
	Pre-op	1 year	Final	Δ Parameter	Pre-op	1 year	Final	Δ parameter	
Segmental angle, degree, mean (SD)	0.54 (3.50)	*5.17* *(2.39)*	4.31 (3.97)	4.66 (3.76)	0.88 (3.21)	*5.35* *(1.98)*	4.29 (3.52)	2.03 (1.16)	.000079^a^*
Translation, mm, mean (SD)	3.62 (1.88)	*1.59* *(1.72)*	1.60 (1.37)	2.02 (1.57)	3.05 (1.59)	*1.85* *(0.92)*	0.95 (1.10)	1.66 (1.39)	.95^a^
Short lumbar lordosis, degree, mean (SD)	14.03 (10.17)	*17.41* *(10.45)*	16.76 (12.74)	2.73 (9.82)	16.66 (12.87)	*17.80* *(10.55)*	17.71 (9.99)	1.05 (9.26)	.28^a^
Whole lumbar lordosis, degree, mean (SD)	28.50 (16.17)	*28.79* *(15.13)*	29.02 (17.04)	0.52 (13.79)	31.43 (16.94)	*32.45* *(14.51)*	33.40 (14.62)	1.97 (12.55)	.50^a^
Pelvic incidence, degree, mean (SD)	54.73 (10.02)	*56.17* *(9.05)*	55.44 (9.08)	− 0.68 (11.21)	55.83 (8.96)	*56.47* *(10.40)*	55.97 (10.26)	− 0.13 (8.08)	.72^a^
Pelvic tilt, degree, mean (SD)	30.80 (9370)	*45.47* *(12.75)*	29.50 (10.41)	− 1.30 (10.79)	28.04 (9.34)	*40.92* *(11.57)*	27.20 (9.16)	− 0.83 (9.20)	.77^a^
Sacral slope, degree, mean (SD)	24.01 (8.20)	*10.70* *(12.84)*	28.84 (8.00)	4.48 (14.24)	28.04 (8.73)	*15.55* *(11.16)*	28.84 (8.75)	− 1.51 (13.58)	.0026^a^*
Thoracic kyphosis, degree, mean (SD)	4.69 (3.69)	*8.69* *(5.43)*	7.99 (3.89)	3.29 (2.18)	4.53 (3.26)	*8.67* *(4.78)*	8.24 (3.71)	3.70 (2.64)	.06^a^
Sagittal vertical axis, cm, mean (SD)	4.10 (1.93)	*4.13* *(1.87)*	4.11 (2.26)	0.01 (2.57)	4.14 (2.33)	*4.41* *(1.88)*	4.13 (2.33)	0.50 (2.10)	.07^a^

^a^Independent Student’s *t* test

*P < .05

*Pre-op* preoperative, *SD* standard deviation

SS also showed a positive correlation with segmental angle correction (Fig. 3e); however, this segmental angle correction did not show a significant positive correlation with PI (Fig. 3f).

### Fusion rate and implant failures

**Fig. 4:**
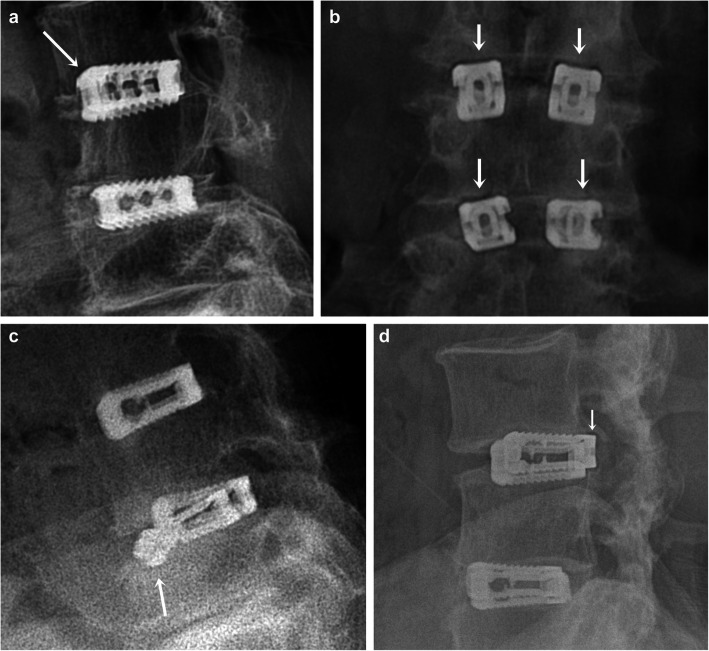
Implant failures after follow-up. **a** Lateral radiograph showing subsidence. **b** Anterior-posterior radiograph showing pseudoarthrosis. **c** Lateral radiograph showing cage breakage. **d** Lateral radiograph showing retropulsion

The implant problems, including cage- and screw-related complications, are summarized in Table 4. The control group showed a high fusion rate than the cage-alone group, but it was not statistically significant (91.51% vs 87.23, *P* = .22). Subsidence (Fig. 4a), pseudoarthrosis (Fig. 4b), cage breakage (Fig. 4c), and retropulsion (Fig. 4) rates were significantly higher in the cage-alone group than in the control group (*P* = .0018, *P* = .02, *P* = .01, and *P* = .01, respectively). However, the rates of PJK and screw malposition were significantly higher in the control group than in the cage-alone group (*P* = .03 and *P* = .02, respectively).

**Table 4:** Comparison of furoin rate and implant failure rate in each group

Implant failure	Stand-alone expandable cage fusion (***n*** = 141 levels)	Screw-assisted fusion (***n*** = 165 levels)	***P*** value
Fusion rate, no. (%)	*123(87.23)*	*151(91.51)*	*.22*^*a*^
Subsidence, no. (%)	*23 (16.31)*	*8 (4.85)*	*.0018*^a^*
Pseudoarthrosis, no. (%)	*13 (9.92)*	*4 (2.42)*	*.02*^a^*
Proximal junctional kyphosis, no. (%, per cases)	7 (10.1)	18 (22.5)	.03^a^*
Cage breakage, no. (%)	*8 (5.67)*	*0 (0)*	*.01*^a^*
Cage retropulsion, no. (%)	*5 (3.55)*	*0 (0)*	*.01*^a^*
Screw malposition, no. (%)	*0 (0)*	*6 (3.63)*	*.02*^a^*

^a^ *χ*^2^ test

*P < .05

*No.* number

## Discussion

**Fig. 5:**
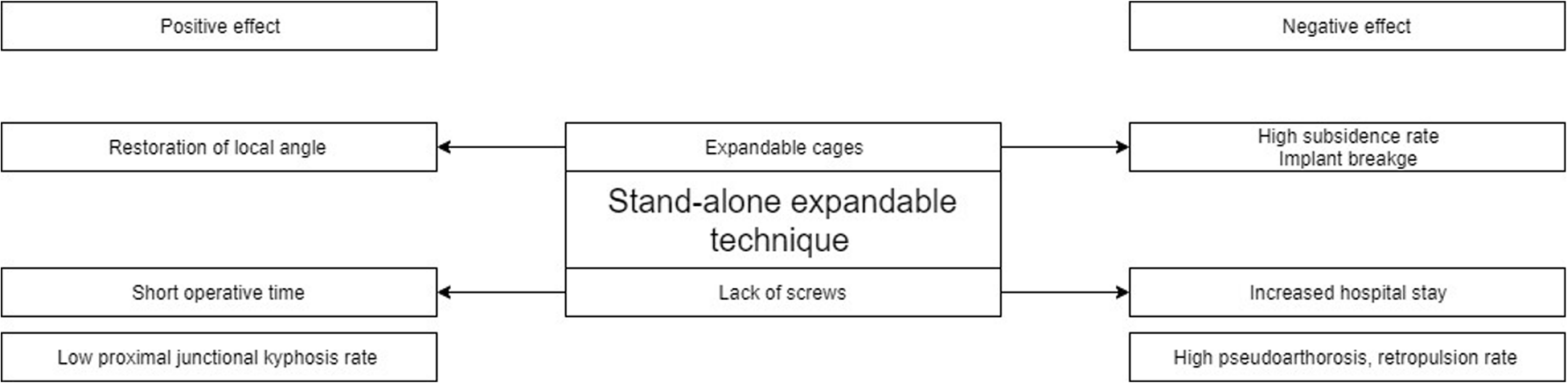
Relationship map of the implant factors, patient factors, and sacropelvic profile

To our knowledge, this study is the first to report the long-term outcomes of posterior stand-alone expandable cage fusion surgery. In this study, insertion of an expandable cage-alone not only increased the segmental angle but also correlated positively with LL. However, LL was not corrected, and the SS increased. High implant failure rates, weak support of the posterior element, and compensatory mechanisms are possible factors affecting these results. On the basis of our results, we have drafted a relationship chart of these factors (Fig. 5).

### Are there any advantages to the posterior stand-alone expandable cage approach?

Screw placement at the pedicle has been regarded as the standard posterior stabilization procedure since 1969, and it was first introduced by Harrington and Tullos [19]. The efficacy and superior support of this technique compared with other techniques, particularly the superior biomechanical strength [20] and presence of three columns, which provide more support than other techniques [21, 22], have been reported. However, this technique needs wide exposure for screw insertion and anatomic landmark confirmation. Furthermore, the reported rate of screw malposition ranges from 0 to 42% [23, 24]. Because we skipped the process of screw placement in this study, the operative time was saved, the paraspinal and posterior facet complex was preserved with a small incision, and radiation exposure was reduced. Compared with the anterior lumbar interbody fusion and lateral lumbar interbody fusion procedures, simultaneous direct decompression can be performed and abdominal organs or hypogastric nerve injury can be avoided [12, 13]. In our result, both the cage-alone and screw fixation groups showed a decrease in the segmental angle in the long term, and expandable cages could achieve a greater angle. This shows that expandable cages have the effect of angle correction locally. In addition, we found that the rate of PJK was significantly lower in the cage-alone group than in the control group. As PJK is induced by overloading the junctional disc space [25], our facet-preserving technique might result in less overloading than the firmly fixed screw technique.

### Is interbody fusion without screw fixation safe?

Compared with other fusion procedures, the possible complications of interbody fusion without screw fixation are totally different. In posterior fusion with a cage, owing to the wide exposure and screw placement, dural tear, rod fracture, PJK, and root damage are common complications [26]. With a stand-alone anterior or oblique approach, insufficient decompression means that additional decompression is required, and psoas muscle weakness and abdominal and vessel injuries [27] are common complications. In the short term, patients who received a posterior expandable cage-alone reported minor complications, such as posterior leg pain, infection, and wound problems [28]. However, long-term complications consisted of implant problems, especially subsidence, pseudoarthrosis, retropulsion, and cage breakage. High subsidence rate and breakage of cage can result from excessive restoration of the local angle [29]. Lack of screw did not maintain stability during the initial period, as shown by the high pseudoartrosis [30] and retropulsion rate. Even though our series showed that implant failure did not need replacement and revisions, it can be the cause of postoperative pain and disability during recovery.

### Why is it that an expandable cage cannot correct LL and spinopelvic profile?

The manufacturers have designed the expandable cages to be able to increase the lordosis by up to 9°; however, our measured mean segmental correction was only 4.66°. Subsidence [29] and pseudoarthrosis [30] are known factors that can reduce the lordotic angle. Cage breakage and retropulsion are possible debilitating events that can decrease LL. This may be the reason why the segmental angle did not correct LL, even though both parameters showed a significant positive relationship. Furthermore, weak posterior fixation can change the sacropelvic profile. In the normal aging process, PT and thoracic kyphosis increase. However, because PI is a consistent parameter [31], SS increases as a compensatory mechanism. However, our results in the cage-alone group were entirely different. Initially, the SS increased more in the cage-alone group than in the control group because of the lack of posterior support. Consequently, the PT was compensated for; hence, PI was preserved. Posterior screw fixation played a role in maintaining the SS in the control group, and the whole spinopelvic profile was better preserved in the control group than in the cage-alone group.

### How to solve issues and gain better outcomes

Three issues should be resolved to achieve better outcomes with this technique. First, we need to use more stable and advanced materials for interbody fusion. The use of enhanced titanium, additional bioactive glass ceramics, and other materials can reduce the rate of pseudoarthrosis [32]. Second, we need to preserve posterior support. Motion-preserving total disc replacement surgery showed more stable outcomes than the currently evaluated method [11] because of complete preservation of the posterior facet complex. Because this method also has a drawback (i.e., it is impossible to decompress the posterior canal), modified minimally invasive techniques, such as unilateral approaches, should be considered. Third, we need to increase bone density. The use of teriparatide in femoral fractures showed efficient prevention of bony subsidence [33]; thus, the use of hormones or medication may play a role in achieving better outcomes.

### Limitations of the study and future scope

This study has limitations and several issues that need to be resolved in future studies. First, given the lack of blinding method and retrospective study design, many patients were lost to follow-up, and many confounding factors by indication are present. Even though we narrowed the indication in multilevel degenerative pathology, misclassification and selection bias can affect operation and follow-up. Second, there may have been major advancements in medications that support bone formation and advancements in the quality of cage materials since the patients in this study were treated. Therefore, it is essential for future studies to address the effects of better bone-forming agents and the application of stronger cage materials. Future studies should also have a multicenter prospective study design.

## Conclusions

We reported the long-term outcomes of posterior expandable cage fusion surgery. The advantages of the procedure include the shorter operative time and low PJK rate. However, the longer hospital stay, higher rates of subsidence and pseudoarthrosis, and ineffective correction of the spinopelvic profile were disadvantages. Stand-alone expandable cage fusion can only restore local balance, but global sagittal balance was not restored. Furthermore, implant safety still has not been proven. This method still needs further investigation in today’s current medical environment of advancements in cage materials and improved medications for bony support.

## Data Availability

We attached all our raw data as a supplementary file.

## Abbreviations

LL: Lumbar lordosis
PJK: Proximal junctional kyphosis
POD: Postoperative day
PI: Pelvic incidence
PT: Pelvic tilt
SS: Sacral slope
